# Acute effects of foam rolling on passive tissue stiffness and fascial sliding: study protocol for a randomized controlled trial

**DOI:** 10.1186/s13063-017-1866-y

**Published:** 2017-03-09

**Authors:** Frieder Krause, Jan Wilke, Daniel Niederer, Lutz Vogt, Winfried Banzer

**Affiliations:** Department of Sports Medicine, Institute of Sport Science, Goethe University Frankfurt, Ginnheimer Landstraße 39, 60487 Frankfurt am Main, Germany

**Keywords:** Foam rolling, Self-myofascial release, Flexibility, Tissue stiffness, Connective tissue, Fascia, Ultrasound, Cross-correlation

## Abstract

**Background:**

Self-myofascial release (SMR) aims to mimic the effects of manual therapy and tackle dysfunctions of the skeletal muscle and connective tissue. It has been shown to induce improvements in flexibility, but the underlying mechanisms are still poorly understood. In addition to neuronal mechanisms, improved flexibility may be driven by acute morphological adaptations, such as a reduction in passive tissue stiffness or improved movement between fascial layers. The aim of the intended study is to evaluate the acute effects of SMR on the passive tissue stiffness of the anterior thigh muscles and the sliding properties of the associated fasciae.

**Methods:**

In a crossover study design, 16 participants will receive all of the following interventions in a permutated random order: (1) one session of 2 × 60 s of SMR at the anterior thigh, (2) one session of 2 × 60 s of passive static stretching of the anterior thigh and (3) no intervention. Passive tissue stiffness, connective tissue sliding, angle of first stretch sensation, as well as maximal active and passive knee flexion angle, will be evaluated before and directly after each intervention.

**Discussion:**

The results of the intended study will allow a better understanding of, and provide further evidence on, the local effects of SMR techniques and the underlying mechanisms for flexibility improvements.

**Trial registration:**

ClinicalTrials.gov, identifier: NCT02919527. Registered on 27 September 2016.

**Electronic supplementary material:**

The online version of this article (doi:10.1186/s13063-017-1866-y) contains supplementary material, which is available to authorized users.

## Background

Self-myofascial release (SMR) is an intensive self-treatment with rigid foam rollers and other small handheld tools based on the exertion of compressive force to the soft tissue. Aiming to tackle dysfunctions of the skeletal muscle and connective tissue, it claims to mimic the effects of manual therapy techniques. Recent studies indicate that SMR, inter alia, improves range of motion (ROM) [1–13] without concurrent decrease in neuromuscular performance [1, 2, 5, 10–12]. In addition to neuronal mechanisms, such as increased stretch tolerance [1, 3, 4], flexibility increases might be attributed to acute morphological adaptations:

First, the fasciae surrounding the muscles of the lower extremity are composed of multiple fibrous layers. Loose connective tissue enriched with hyaluronic acid [14, 15] allows these layers to slide against each other during motion (e.g., contraction or elongation of the underlying muscle) [14]. Several authors assume a positive effect of SMR on fascial sliding properties, e.g., through breaking up adhesions or loosening cross-links [10, 16].

Another hypothesized morphological consequence of SMR is the alteration of passive tissue stiffness, as occurs after static stretching [17–22]. A plethora of studies have demonstrated the existence of myofibroblasts (and their ability to impact stiffness) in fascia [23, 24]. Moreover, according to in vitro experiments, fascial hydration has been shown to alter biomechanical tissue properties [25]. Compression of the muscle and the surrounding fascial tissue (as occurs by the use of a foam roller) might hence stimulate contractile cell activity, affect tissue hydration [10, 16] and microarchitecture of cell cytoskeleton [26] or muscle filament mechanical properties [27] and thereby alter tissue stiffness. Although these mechanisms seem plausible, there is no scientific evidence for these assumptions. Most studies focus solely on functional parameters (e.g., flexibility, strength, recovery) in practice-based settings. However, knowledge of the underlying physiological processes would allow a more effective selection of therapeutic and performance-related indications. The aim of the intended study is to evaluate the acute effects of SMR on the passive tissue stiffness of the anterior thigh muscles and the sliding properties of the associated fasciae. We hypothesize, that (1) SMR is able to decrease passive stiffness in the same manner as static stretching [17–22], that (2) increased interlayer sliding of fascial layers occurs following treatment and (3) that these processes are associated with an increase in joint flexibility.

## Methods

### Study design

The study will adopt a randomized crossover design. After signing informed consent prior to study enrollment, healthy participants receive all of the following interventions in a permutated random order:

1. One session of 2 × 60 s of SMR at the anterior thigh
2. One session of 2 × 60 s of passive static stretching of the muscles at the anterior thigh
3. No intervention

**Fig. 1:**
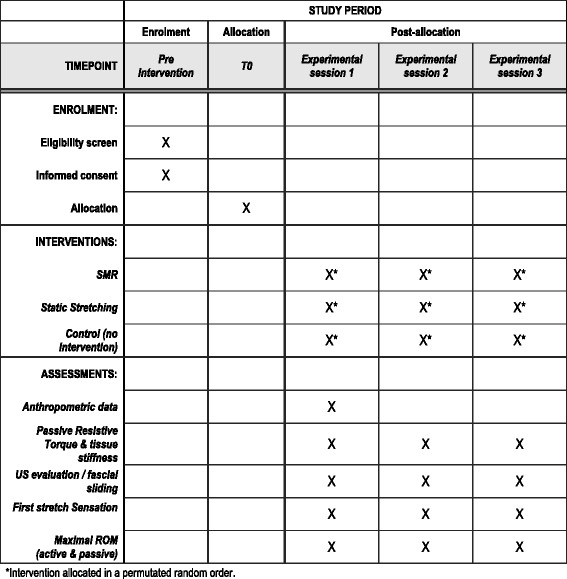
Standard Protocol Items: Recommendations for Interventional Trials (SPIRIT) figure

At least 2 days prior to the experimental conditions, participant receive a standardized familiarization session including the testing procedure and an introduction to the SMR intervention to minimize learning effects. Before each intervention, main outcomes are measured. Initial data collection is followed by a 15-min passive break to prevent the measurement procedure to overlie possible treatment effects. Immediately after the intervention or control condition, post-intervention outcome parameters are collected (see Additional file 1 and Fig. 1). All experimental trials will be performed at the same time of day (±2 h) for each subject. A period of at least 3 days serves as a wash-out phase between the three experimental testing sessions [22].

The study was approved by the local Ethics Committee of the Faculty of Psychology and Sport Science (Goethe-University Frankfurt). The collection, transfer, storing and analyses of personal data during the trial is in accordance with applicable law. Data are collected at the Department of Sports Medicine, recorded on paper or electronically and treated confidentially. Data transfer requires pseudonymization and is restricted to the principal investigator, study physicians and the competent Ethics Committee for the assessment of study results and adverse events.

The protocol was written in accordance to the Standard Protocol Items: Recommendations for Interventional Trials (SPIRIT) guidelines, a copy of the SPIRIT Checklist has been included as Additional file 2.

### Inclusion/exclusion criteria

Subjects are eligible for the trial if they meet the following criteria:

- Age between 20 and 40 years
- No history of orthopedic injuries in the lower extremity in the last 12 months

Subjects are ineligible if they have any of the following criteria:

- Any history of psychiatric, cardiovascular, endocrine, neurological or metabolic disorders
- Any current medication that might affect pain perception or proprioception
- Muscle soreness
- Pregnancy/nursing period
- Nonspecific musculoskeletal disorders

### Sample size calculation

To determine the required sample size, an a priori sample size calculation was performed using G*Power (G*Power, Version 3.1, Heinrich-Heine University Düsseldorf, Germany). Based on previous studies on the effect of static stretching on tissue stiffness [21], we expect a medium effect (f^2^ = 0.25; *α* = .05 and *β* = .80). Considering the omnibus testing (2 × 3 repeated measurements ANOVA/Friedman test), calculated sample size was *n* = 42. A 10% dropout rate assumed and taking into account our crossover study design, the sample size to be recruited is *n* = 16 participants.

### Randomization

A balanced permutation randomization sequence of treatment orders is generated using an electronic randomization algorithm (www.randomization.com). Body side of treatment is randomized using the same algorithm. Randomization is equivalent to the order of study inclusion.

### Interventions

Both interventions consist of two 60-s bouts of either dynamic SMR with a foam roller or static stretching of the myofascial tissue of the anterior thigh. The SMR intervention is performed in the prone position. The participants are instructed to place their body weight on a polypropylene foam roller with a length of 30 cm and a diameter of 15 cm (Blackroll, Bottighofen, Switzerland). Applying pressure to the tissue of the anterior thigh, they perform a rolling motion from the proximal aspect of the thigh (inferior to the anterior superior iliac spine) to the knee (see Additional file 3). Once the foam roller reaches the superior border of the patella, participants are instructed to return to the starting position and continue the sequence for the remainder of the 60 s [5, 7]. The rolling frequency is standardized using a metronome set at 60 beats per minute (bpm). Participants are instructed to roll at a velocity of two metronome beats (thus, 2 s) for each rolling direction, resulting in 15 complete rolling cycles in 60 s (0.25 Hz). Intensity of pressure is controlled subjectively with a target Numerical Rating Scale (NRS) rating of 7/10 (0 representing no discomfort and 10 representing maximal discomfort) during the intervention. After a 30-s break in a relaxed prone position, participants perform a second bout.

Similar to SMR, also passive static stretching of the anterior thigh muscles is performed in the prone position with a pre-stretch of the hip (200° in total) using a bed wedge with a 20° inclination. In this position, the investigator performs a passive static stretching maneuver by manually flexing the knee of the subject while continuously controlling for secondary movement of the lumbar spine. Stretch intensity is adjusted according to the feedback of the subject (target NRS rating of 7/10). The position is held for 60 s, followed by a 30-s rest in a relaxed position and a second bout of stretching at the same target intensity for 60 s.

### Outcomes

#### Main outcomes

##### Passive resistive torque (PRT)

Passive resistive torque of the quadriceps muscle-tendon unit is evaluated using a computerized isokinetic dynamometer (Biodex system 3 Pro, Biodex Medical, Shirley, NY, USA). The participant is placed in a standardized position on the seat of the dynamometer (see Additional file 4). The pelvis as well as the thigh of the tested leg are fixed with restriction straps to minimize secondary movement. The opposite hip is fixed at 90° flexion to limit pelvic and lumbar motion. The knee axis is aligned with the rotational axis of the dynamometer.

To obtain PRT, the lower leg is moved from full knee extension (0°) to maximal achievable knee flexion angle with an angular velocity of 5°/s in passive mode of the dynamometer. Torque (T) and angle (θ) are recorded at 100 Hz. This procedure has been described as a reliable method to evaluate passive tissue properties for various positions and muscles (intraclass correlation coefficient (ICC) ranging from 0.88 to 1.00) [28–33]. Torque data is gravity corrected and filtered using a Butterworth, zero-lag, fourth-order low-pass filter with a 10-Hz cutoff frequency [22].

##### Tissue stiffness

A fourth-order polynomial (FOP) model is fitted on the T-θ data (and stiffness is calculated using the slope of the FOP model [34, 35]. Passive resistive torque as well as stiffness values from four angles during the last 13° of passive tissue tensioning (1°, 5°, 9° and 13°) are calculated and serve as a quantification of resistance and stiffness during passive motion [22].

To monitor muscle activity, surface electromyography (sEMG) is used with two surface electrodes (Ambu Blue Sensor, Ambu GmbH, Bad Nauheim, Germany) placed on the head of the M. rectus femoris muscle with an 8-mm inter-electrode distance and one reference electrode on the patella, according to SENIAM recommendations [36]. Participants are provided with live biofeedback of muscle activity to prevent involuntary muscle contraction.

##### Fascial sliding

While assessing PRT, the probe of a high-resolution ultrasound (US) device (Siemens Acuson X300, Siemens Healthcare GmbH, Erlangen, Germany) is positioned on the thigh (for details, see below). Sliding of fascial layers is quantified with a frame-by-frame, cross-correlation algorithm of the generated US images obtained during the passive movement. The cross-correlation method developed in MATLAB (The MathWorks, Inc, Natick, MA, USA) by Dilley and colleagues [37] is used to calculate the correlation coefficient between the pixel gray levels for selected rectangle-shaped regions of interest (ROIs) in two adjacent images. The pixel shift providing the maximum correlation coefficient corresponds to the relative movement between two frames [37]. The method has been extensively used to quantify nerve movement and represents a reliable method to quantify tissue movement in vivo (ICC ranging from 0.70 to 0.99) [37–44].

The linear array US transducer used (4–11.4-MHz, 38.4-mm footprint) is placed on the proximal third of the muscle belly of the M. rectus femoris and sequences of 20 s are captured at 10 frames/s during passive knee flexion (starting at 0° until 100° of knee flexion at 5°/s). US transducer location is marked on the skin with a permanent marker. Participants are instructed to renew the marker on a daily basis to ensure equal transducer placement at all three testing sessions. Six ROIs are placed on the superficial and deep layers of the fascia lata, respectively, to quantify sliding of these layers during passive stretching of the underlying muscle (see Additional file 5). Maximal lateral movement of ROIs/fascial layers is calculated and analyzed as a quantification of fascial sliding.

#### Secondary outcomes

##### Flexibility and ROM

Three parameters representing flexibility and ROM are assessed. The position of the first stretch sensation is quantified using the isokinetic dynamometer in the above-described position. In passive mode, the knee is flexed from full extension to flexion at 5°/s. The subject uses a switch to stop the passive movement at the position of the first stretch sensation.

Maximal active as well as passive knee flexion ROM in the sagittal plane is assessed in prone lying with a 3D ultrasonographic movement analysis system (zebris CMS20, zebris Medical GmbH, Isny, Germany). Inter- as well as intra-rater reliability have been described as good to excellent (*r* between .84 and .96) [45]. A triplet of ultrasonographic markers is placed on the lower leg, a second triplet is placed as a reference on the thigh. In this position, participants are instructed to perform three consecutive active knee flexion-extension cycles at a self-selected velocity. Subsequently, the investigator performs three passive knee flexion-extension cycles. Movements are recorded in three dimensions at 20 Hz, and maximal active as well as passive knee flexion ROM in the sagittal plane can be calculated as the maximal displacement relative to the starting position recorded by the US markers.

### Statistical analysis

The software SPSS (version 22.0, SPSS Inc., Chicago, IL, USA) is used for statistical analyses. All calculation are performed after checking the underlying assumptions for (1) parametric or (2) nonparametric testing. Statistically significant differences between pre and post measurements, as well as the three conditions (SMR, stretching, control), are tested with (a) a 2 × 3 repeated measurements ANOVA followed by LSD post hoc analyses or (b) Friedman test followed by Dunn-Bonferroni tests. Associations between measures of tissue stiffness, respectively fascial sliding and ROM measures are evaluated with (a) Pearson or (b) Spearman correlation analyses. The level of statistical significance is set to *α* < 0.05. Due to the explorative design of the study, an alpha-level correction will not be performed for the multiple hypotheses testing.

## Discussion

The use of foam rollers in the context of treatment and training of fascial tissues has gained considerable popularity in the last decade. Current research suggests that SMR techniques can improve ROM, but the effects on passive tissue stiffness and fascial sliding as possible influencing factors have not been evaluated yet.

Knowledge about these effects will allow a better understanding of, and provide further evidence on, the local effects of SMR techniques and the factors leading to improvements in ROM. In contrast to static stretching, foam rolling does not seem to negatively affect neuromuscular performance, so the acute biomechanical mechanisms for increases in flexibility might differ.

The results of the intended study will have several implications for clinical practice and the implementation of SMR techniques into therapy and training. Knowledge of the effect on passive tissue stiffness and fascial sliding allows the clinician to determine the optimal timing for the use of SMR during warm-up before competition or during therapy to restore normal ROM.

## Trial status

At the time of submission of this manuscript, recruitment is ongoing.

## Additional files

Additional file 1: Study flow chart. (JPG 2046 kb)
Additional file 2: SPIRIT Checklist. (DOC 120 kb)
Additional file 3: Illustration of the self-myofascial release (SMR) intervention. (JPG 2832 kb)
Additional file 4: Positioning of participants on the isokinetic dynamometer. (JPG 1897 kb)
Additional file 5: Exemplary (based on a pilot participant) visualization of the results of the Cross-correlation analysis. Description of data: *blue boxes*: individual regions of interest (ROIs), *white rectangles*: mean movement of all ROIs, *yellow rectangles*: movement of individual ROIs. ROIs at the beginning of the passive knee flexion (A). Motion of the ROIs at the end of the passive knee flexion (B). Resulting line graph of motion of separate ROIs (C). (JPG 2079 kb)

## Abbreviations

bpm: Beats per minute
FOP: Fourth-order polynomial
NRS: Numerical Rating Scale
PRT: Passive resistive torque
ROM: Range of motion
sEMG: Surface electromyography
SMR: Self-myofascial release
SPIRIT: Standard Protocol Items: Recommendations for Interventional Trials
T: Torque
