# Supplementary figures and images for: Acute effects of foam rolling on passive tissue stiffness and fascial sliding: study protocol for a randomized controlled trial
Source: Trials. 2017 Mar 9;18:114. doi: 10.1186/s13063-017-1866-y (PMC5343315; doi:10.1186/s13063-017-1866-y)

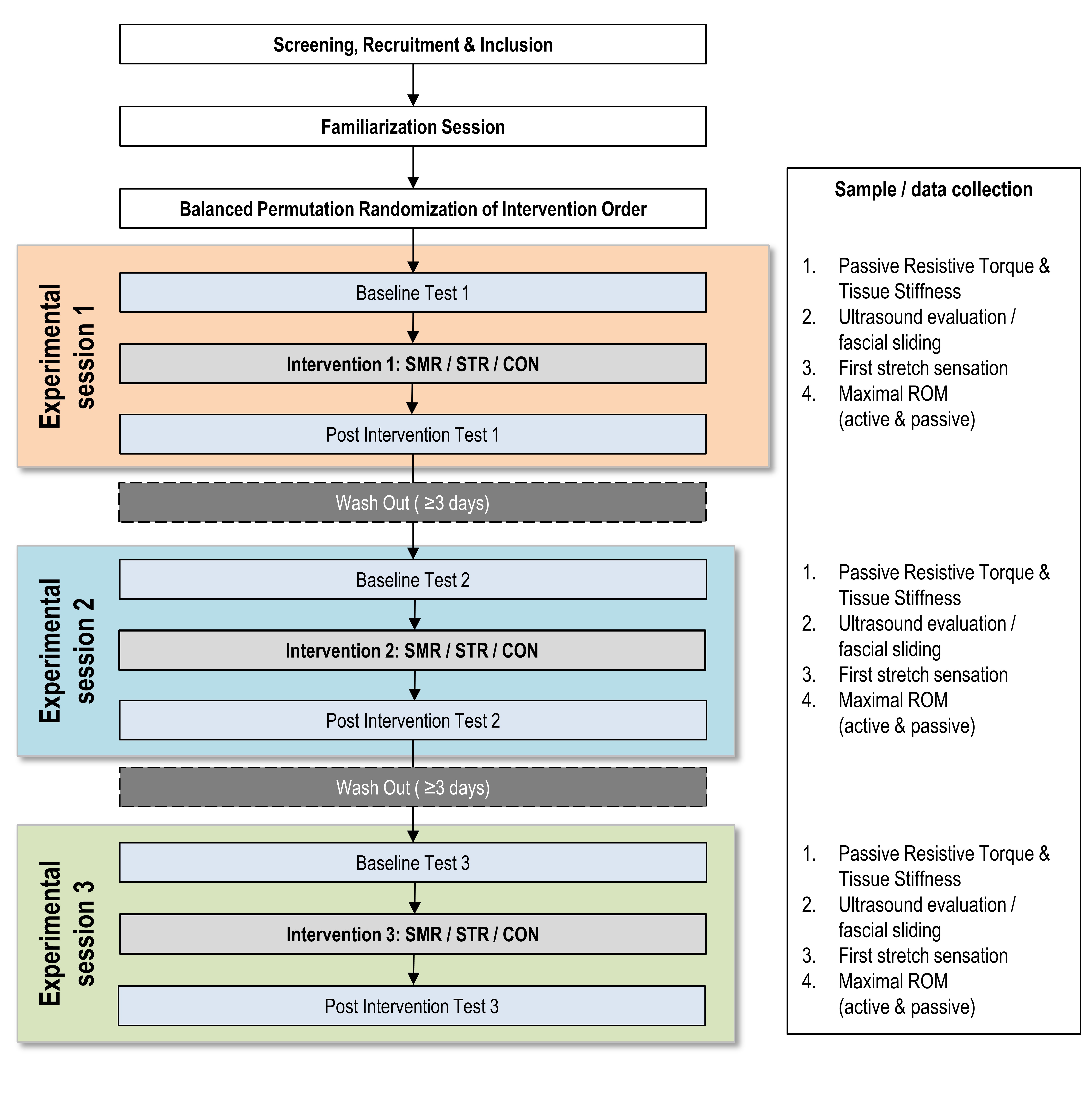

Supplement: Additional file 1: — Study flow chart. (JPG 2046 kb) [file 13063_2017_1866_MOESM1_ESM.jpg]

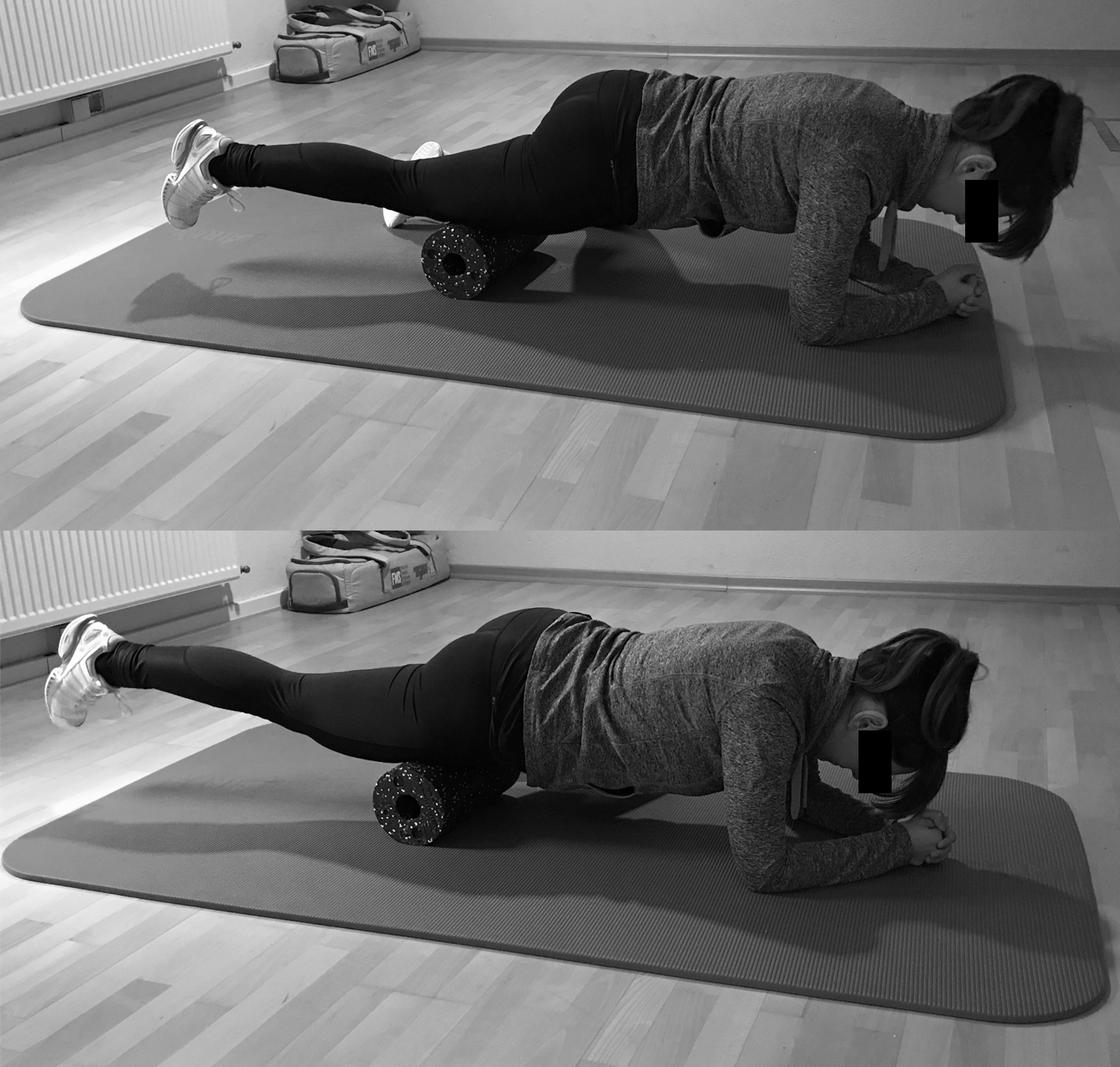

Supplement: Additional file 3: — Illustration of the self-myofascial release (SMR) intervention. (JPG 2832 kb) [file 13063_2017_1866_MOESM3_ESM.jpg]

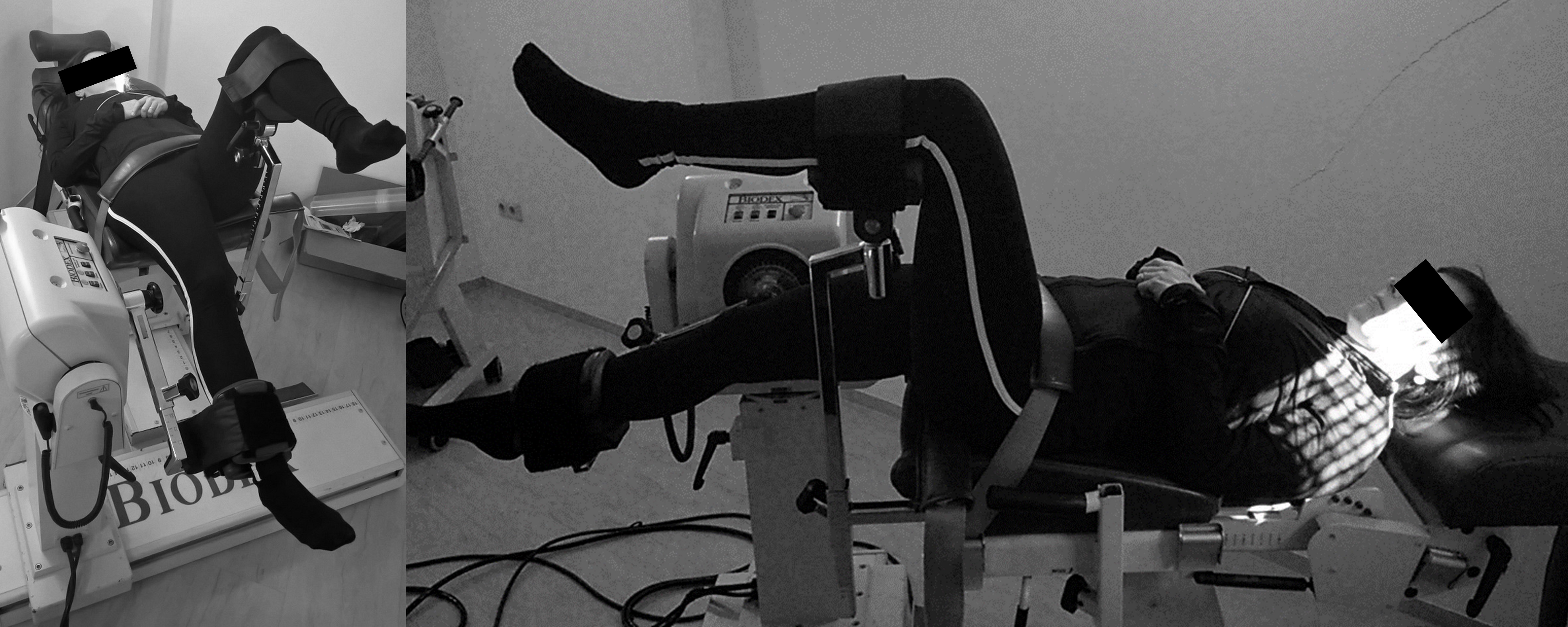

Supplement: Additional file 4: — Positioning of participants on the isokinetic dynamometer. (JPG 1897 kb) [file 13063_2017_1866_MOESM4_ESM.jpg]

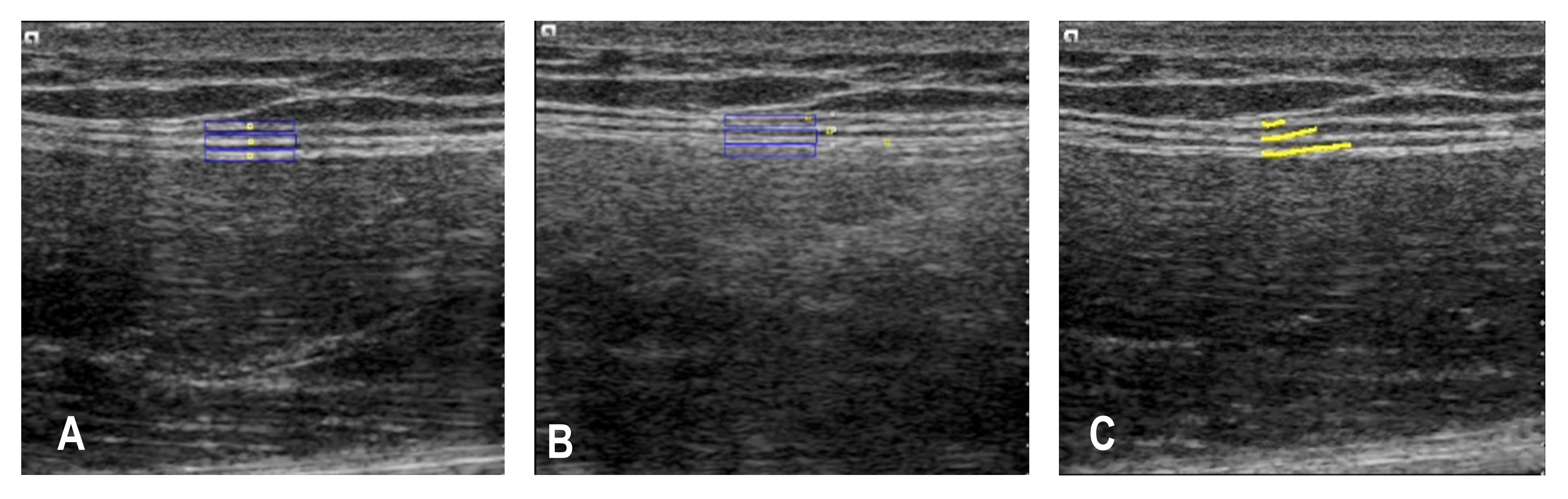

Supplement: Additional file 5: — Exemplary (based on a pilot participant) visualization of the results of the Cross-correlation analysis. Description of data: blue boxes: individual regions of interest (ROIs), white rectangles: mean movement of all ROIs, yellow rectangles: movement of individual ROIs. ROIs at the beginning of the passive knee flexion (A). Motion of the ROIs at the end of the passive knee flexion (B). Resulting line graph of motion of separate ROIs (C). (JPG 2079 kb) [file 13063_2017_1866_MOESM5_ESM.jpg]
